# First Complete Genome Sequence of Cucurbit aphid-borne yellows virus from Papua New Guinea

**DOI:** 10.1128/genomeA.00162-18

**Published:** 2018-03-15

**Authors:** Solomon Maina, Martin J. Barbetti, Owain R. Edwards, David Minemba, Michael W. Areke, Roger A. C. Jones

**Affiliations:** a School of Agriculture and Environment, Faculty of Science, The University of Western Australia, Crawley, Western Australia, Australia; b Institute of Agriculture, Faculty of Science, The University of Western Australia, Crawley, Western Australia, Australia; c Cooperative Research Centre for Plant Biosecurity, Canberra, Australian Capital Territory, Australia; d CSIRO Land & Water, Floreat Park, Western Australia, Australia; e Kana Aburu Haus Sir Alkan Tololo Research Centre, PNG National Agriculture Research Institute, Lae, Morobe Province, Papua New Guinea; f National Agriculture Quarantine and Inspection Authority, Port Moresby, National Capital District, Papua New Guinea; g Department of Primary Industries and Regional Development, South Perth, Western Australia, Australia

## Abstract

Analysis of an RNA-Seq library from cucumber leaf RNA revealed the first complete genome sequence of Cucurbit aphid-borne yellows virus (CABYV) from Papua New Guinea. We compared it with 36 complete CABYV genomes from other world regions. It most resembled the genome of South Korean isolate GS6.

## GENOME ANNOUNCEMENT

As part of a biosecurity project to examine possible connectivity between viruses infecting crops in northern Australia and nearby countries, virus genomes from East Timorese and Australian plant samples were compared (1–14). Agriculture was first practiced in Papua New Guinea (PNG) around 6,000 years ago (15), but in Australia it has only been practiced for 229 years, and in remote regions, agriculture began as recently as 60 years ago. During October 2016, 23 leaf samples were collected from cucurbit plants with virus-like symptoms growing in PNG. These samples were blotted onto fast technology for analysis of nucleic acids (FTA) cards before dispatch to Australia, where they were subjected to next-generation sequencing. A complete genome of Cucurbit aphid-borne yellows virus (CABYV) was obtained from cucumber (Cucumis sativus) sample 10PN from Zage Village, Goroka, Eastern Highlands Province, PNG. CABYV was first described in 1992 causing yellow leaf symptoms in cucurbit plants in France (16) and belongs to genus Polerovirus, family Luteoviridae (17). CABYV is a phloem-limited virus transmitted persistently by aphids, including Aphis gossypii and Myzus persicae (16). It consists of a single-stranded positive-sense RNA molecule with a length of 5.7 kb (17). In sample 10PN, CABYV was detected by analysis of nonpolyadenylated transcripts derived from RNA-Seq stranded libraries (1–14) prepared from RNA extracted from FTA cards (1–14, 18–21).

Total RNA was extracted from all 23 leaf samples preserved on the FTA cards from PNG and subjected to library preparation as described previously (1–14). The libraries were sent to Macrogen, Inc., South Korea, where they were subjected to HiSeq 2500 sequencing using a TruSeq Rapid SBS kit v4 (Illumina) with 151 cycles to generate paired-end reads. Reads were assembled and genomes annotated using CLC Genomics Workbench 6.5 (CLC bio) and Geneious 8.1.7 (Biomatters) (1–14, 22, 23).

CABYV isolate 10PN yielded 19,041,390 reads and, after trimming, 19,038,589 reads remained. *De novo* assembly generated 1,777 contigs and 1,418,175 reads mapped to the contigs of interest with 24,776× coverage. The genome obtained had 5,671 nucleotides (nt) and six open reading frames (ORFs) organized into two clusters, as with other poleroviruses (24). A BLAST-based search and nucleotide analysis (25) revealed that isolate 10PN most resembled South Korean isolate GS6 (KR231949), with 89.0% nt identity. The percent nt identity between East Timorese CABYV isolate AL50 (KY617826) (8) and isolate 10PN from PNG was 88.0%, indicating lack of genetic connectivity. When 10PN’s P3 gene sequence was aligned with five CABYV-like P3 gene sequences from Tasmania, Australia, currently available in GenBank, it most closely resembled isolate 18 (GenBank accession number HQ543088), with 81.5% nt identity, which again indicates lack of genetic connectivity. Future sequencing of CABYV in Tasmania and mainland Australia is needed to obtain complete genomes to compare with CABYV genomes from PNG and other neighboring countries.

### Accession number(s).

The complete genome sequence of CABYV isolate 10PN has been deposited in GenBank with the accession number MG780352.
